# Supplementary figures and images for: Measurement of Glutamate Suppression in a 6-OHDA-Induced Dopamine Deficiency Rat Model Following Acute Single-Dose L-DOPA Using GluCEST/MRS
Source: Biomedicines. 2025 Nov 12;13(11):2761. doi: 10.3390/biomedicines13112761 (PMC12650681; doi:10.3390/biomedicines13112761)

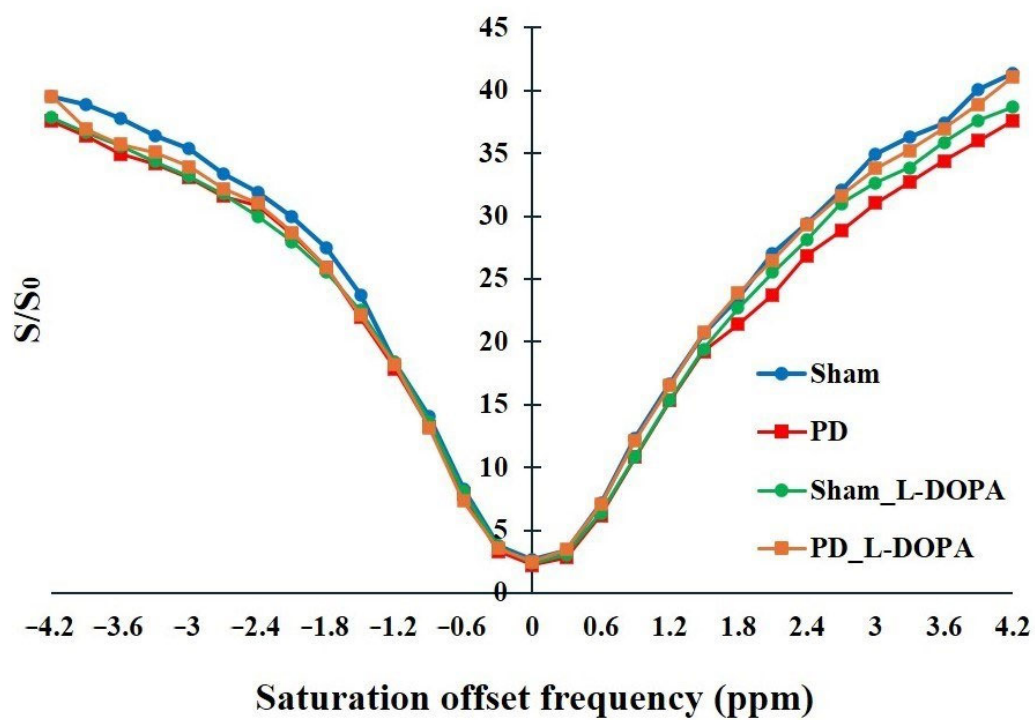

**Figure S1.** The cumulative curves of Z-spectrum.

Supplement: Supplementary file 1 [file biomedicines-13-02761-s001.zip › biomedicines-3912566-supplementary.pdf]
